# Do non-mammary conditions influence patients’ cosmetic perception after breast conserving surgery?

**DOI:** 10.3389/fonc.2024.1432206

**Published:** 2025-01-28

**Authors:** Idam de Oliveira-Junior, Fabíola Cristina Brandini da Silva, Almir José Sarri, René Aloísio da Costa Vieira

**Affiliations:** ^1^ Postgraduate Program of Oncology, Barretos Cancer Hospital, São Paulo, Brazil; ^2^ Nucleous of Mastology, Barretos Cancer Hospital, São Paulo, Brazil; ^3^ Postgraduate Program of Tocogynecology, Botucatu Medical School, São Paulo State University, UNESP, São Paulo, Brazil

**Keywords:** breast cancer, conservative surgery, breast-conserving surgery, oncoplastic surgery, cosmesis, quality of life

## Abstract

**Introduction:**

Compared to mastectomy, breast-conserving surgery (BCS) guarantees equivalent local control and survival, with lower morbidity and better quality of life (QOL), even in the long term. However, some BCS patients consider the cosmetic result to be unsatisfactory, which may affect QOL.

**Material and methods:**

This prospective, cross-sectional study included patients who underwent BCS. The patients answered the European Organization for Research and Treatment of Cancer Quality of Life questionnaire (EORTC QLQ) - C30, EORTC QLQ-BR23 and Breast Cancer Treatment Outcome Scale (BCTOS) questionnaires, underwent cosmetic breast self-assessment and had their breasts photographed. The photographs were analyzed using Breast Cancer Conservative Treatment. Cosmetic results (BCCT.core). For the categorical variables, the frequencies were calculated; for the numerical variables, the mean and standard deviation. The BCCT.core results were compared with the cosmetic results of the patients, which yielded four possible results: concordant satisfaction, discordant satisfaction, concordant dissatisfaction and discordant dissatisfaction (satisfactory BCCT.core evaluation but patient dissatisfaction). The kappa test was used for agreement between categorical variables. Student’s t test and Mann-Whitney were used to assess the relationship between QOL and cosmetic results. The ANOVA were performed with the adjusted Bonferroni correction to compare the four groups.

**Results:**

A total of 300 patients were evaluated, 298 underwent self-assessment of the breasts (76.8% satisfactory results and 23.2% unsatisfactory) and 297 underwent BCCT.core evaluation (29.9% satisfactory results and 79.1% unsatisfactory), which had a kappa of 0.095 (p = 0.01). In the self-assessment, patients with unsatisfactory cosmetic results had worse overall health, physical, functional, emotional, cognitive, and social capacity, fatigue, pain, dyspnea, financial difficulties, body image; future prospects, side effects, breast symptoms, functional aspects, cosmetics and edema. When we used software evaluation, these relationships did not have the same proportions. In patients with “discordant dissatisfaction”, higher pain scores and worse functionality on the treated side were found.

**Conclusion:**

An unsatisfactory cosmetic result was associated with worse QOL, which may be associated with other factors, such as breast pain and functionality.

## Introduction

With the increased in survival, the evaluation of breast cancer surgical treatment has gone beyond pure oncological analysis. Quality of life (QOL) and cosmetic outcomes have become an emphasis, not only for the patients but also professionals involved in the treatment (1).

When compared to mastectomy, breast-conserving surgery (BCS) guarantees equivalent local control and survival, with lower morbidity and higher QOL, even in the long term. However, factors such as age, tumor size, and body mass index may negatively affect the cosmetic outcomes of breast conservation, potentially influencing the QOL of survivors (2).

There is no standard method for the evaluating this cosmetic result, which can be evaluated using objective and subjective methods, but there is low agreement between them (3, 4). Although many patients evaluate their cosmetic outcomes better than objective methods and breast surgeons do, a portion of patients are dissatisfied (5) and require reconstructive surgery after BCS (6). The major problem is when the patient reports dissatisfaction, despite good results determined by health professionals and/or objective methods.

In this sense, given the scarcity of literature, it is necessary to understand the relationship between the late cosmetic outcome of the breasts and the patients’ QOL and the potential associated factors.

## Material and methods

This was a prospective, cross-sectional study approved by the research ethics committee under number 782/2014, with grant FAPESP (2014/08197-0). Patients in follow-up at the Mastology and Breast Reconstruction outpatient clinic of the Barretos Cancer Hospital, who underwent BCS for breast cancer, were randomly included after signing the informed consent form.

Female patients, at least one year after the end of radiotherapy, without metastatic disease and/or locoregional recurrence were included. Those with cognitive limitations for cosmetic self-assessment and answering the quality-of-life questionnaires, in addition to those with bilateral breast cancer were excluded.

The patients filled out the European Organization for Research and Treatment of Cancer Quality of Life questionnaire (x QLQ) - C30, the EORTC QLQ-BR23 and Breast Cancer Treatment Outcome Scale (BCTOS). They self-assessed the cosmetic outcome of the breast (excellent, good, fair, poor) and their breasts were photographed in a standardized way (1-meter distance with a point marked on the sternal notch and another 20 cm below, at the sternal level, for distance calibration). The photographs were analyzed prospectively, transversally and blindly using Breast Cancer Conservative Treatment Cosmetic results (BCCT.core software) - excellent, good, fair, poor. The data from the medical records were evaluated retrospectively in a standardized way. Cosmetic results classified as excellent and good were considered satisfactory. Those classified as fair and poor were categorized as unsatisfactory.

Furthermore, four different scenarios were analyzed: concordant satisfaction (satisfactory cosmetic results both from the software and patient viewpoints), discordant satisfaction (unsatisfactory results according to the software and satisfactory results according to the self-assessment), concordant dissatisfaction (unsatisfactory cosmetic results according to both the software and self-assessment) and discordant dissatisfaction (satisfactory results according to the software and unsatisfactory results according to the self-assessment).

For the categorical variables, the frequencies (absolute and relative) were calculated; for the numerical variables, the mean and standard deviation. To evaluate the agreement between the categorical variables, the kappa test was used. To quantify the relationship between QOL and cosmetic results, Student’s t test was used for normally distributed variables and the Mann-Whitney test for nonparametric variables. Analysis of variance (ANOVA) was carried out for the difference between the type of satisfaction and quality of life, and the Bonferroni corrected test was used to analyze the differences. The analyses were performed using IBM-SPSS software, version 27.0, and the significance level adopted was 5%.

## Results

A total of 300 patients (76% who underwent classical BCS and 24% who underwent oncoplastic surgery) were evaluated, a series described in a previous study validating the BCTOS questionnaire (7). Among them, 298 self-assessed their breasts (76.8% satisfactory result and 23.2% unsatisfactory), and 297 underwent BCCT.core evaluation (29.9% satisfactory result and 79.1% unsatisfactory), with a kappa of 0.095 (p 0.01). The mean age of the patients was 58.8 years (25.6-87.5; standard deviation – SD 9.6), the mean tumor size was 2.2 cm (1.2-20.6; SD 1.4), the mean education level was 7.3 years (0–33; SD 5.3) and the mean follow-up time from the first medical evaluation to participation in the study was 7.4 years (1.2–20.6; SD 4.3).

According to satisfaction with the breast results (**Table 1**) in the EORTC-C30 questionnaire, the dissatisfied patients had worse indices of global health and physical, functional, emotional, cognitive and social capacity. Regarding symptoms, they had higher rates of fatigue, pain, dyspnea and financial difficulties. From the EORTC-BR23 results, worse body image, future prospects and more side effects and breast symptoms were observed. According to the BCTOS, in which higher values correspond to greater differences and worse results between the two breasts, worse functional and cosmetic aspects, breast pain, and edema were found. Multiple non-breast features were related to unsatisfactory results. However, under the objective analysis of the software, these relationships did not present statistical significance in the same proportions. In dissatisfied patients, lower physical capacity and sexual pleasure and worse cosmetic outcomes and edema, were observed.

**Table 1:** Quality of life depending on the results observed by patients and software*.

Questionnaire	Domain	Patient self-assessment			BCCT.core		
		Satisfactory results	Unsatisfactory results	p	Satisfactory results	Unsatisfactory results	p
EORTC	Global Health	79.91 (21.54)	72.71 (21.38)	0.005	79.59 (19.74)	77.68 (22.52)	0.729
QLQ C30	Physical capacity	81.69 (17.56)	73.41 (19.02)	0.001	84.12 (16.07)	77.88 (18.82)	0.004
	Functional capacity	82.97 (25.24)	73.67 (27.92)	0.009	84.46 (23.81)	79.41 (27.01)	0.102
	Emotional Ability	66 (29.4)	46.01 (35.37)	<0.001	63.86 (31.03)	60.4 (32.21)	0.393
	Cognitive ability	71.11 (30.88)	60.63 (34.65)	0.017	69.66 (32.62)	68.43 (31.72)	0.639
	Social capacity	93.45 (17.12)	86.71 (23.67)	0.031	91.95 (18.48)	91.83 (19.31)	0.766
	Fatigue	18.44 (24.33)	31.88 (27.87)	<0.001	20.22 (23.6)	21.69 (26.23)	0.714
	Nausea and vomiting	5.02 (13.07)	8.21 (18.44)	0.184	4.68 (11.78)	6.17 (15.56)	0.718
	Pain	28.6 (30.56)	38.41 (33.25)	0.03	27.53 (27.65)	32.13 (32.85)	0.456
	Dyspnoea	8.3 (21.04)	16.91 (31.11)	0.034	6.74 (16.8)	11.7 (26.35)	0.315
	Insomnia	27.8 (36.79)	37.68 (41.58)	0.059	26.97 (35.84)	31.41 (39.13)	0.423
	Appetite loss	9.32 (24.79)	15.69 (30.18)	0.115	7.95 (20.83)	11.54 (27.52)	0.415
	Constipation	22.27 (34.54)	22.71 (36.82)	0.928	20.97 (34.22)	23.08 (35.46)	0.552
	Diarrhoea	5.68 (20.28)	11.11 (27.22)	0.128	4.87 (19.82)	7.85 (23.11)	0.164
	Financial difficulties	14.26 (31.07)	25.6 (39.25)	0.030	14.98 (31.39)	17.63 (34.35)	0.644
EORTC	Body image	86.39 (21.10)	65.58 (34.83)	<0.001	83.9 (26.61)	80.57 (26.34)	0.109
QLQ BR23	Sexual functioning	76.49 (25.01)	70.05 (28.52)	0.071	75.09 (25.02)	74.76 (26.82)	0.904
	Sexual enjoyment	45.95 (29.93)	45.95 (29.76)	0.999	52.71 (29.31)	42.71 (29.7)	0.05
	Future perspective	57.64 (40.05)	46.86 (39.74)	0.05	57.68 (37.53)	54.01 (41.38)	0.532
	Side effects	22.27 (18.78)	33.4 (22.79)	<0.001	21.94 (17.75)	25.96 (21.22)	0.223
	Breast symptoms	17.36 (20.02)	29.23 (26.95)	<0.001	19.1 (23.04)	20.63 (22.1)	0.474
	Arm symptoms	26.25 (27.32)	31.88 (29.64)	0.142	23.97 (26.05)	29.06 (28.68)	0.125
	Hair loss	24.44 (36.31)	37.04 (42)	0.120	30.08 (39.3)	27.06 (37.97)	0.802
BCTOS	Functional	1.71 (0.76)	2.09 (0.9)	0.002	1.74 (0.77)	1.83 (0.83)	0.451
	Cosmetic	2.07 (0.7)	2.77 (0.72)	<0.001	1.93 (0.67)	2.37 (0.76)	<0.001
	Breast-specific pain	1.83 (0.82)	2.39 (0.98)	<0.001	2.04 (0.9)	1.94 (0.89)	0.378
	Oedema	1.53 (0.67)	1.84 (0.78)	0.001	1.47 (0.68)	1.65 (0.72)	0.013

*Mean and standard deviation.

**Figure 1:**
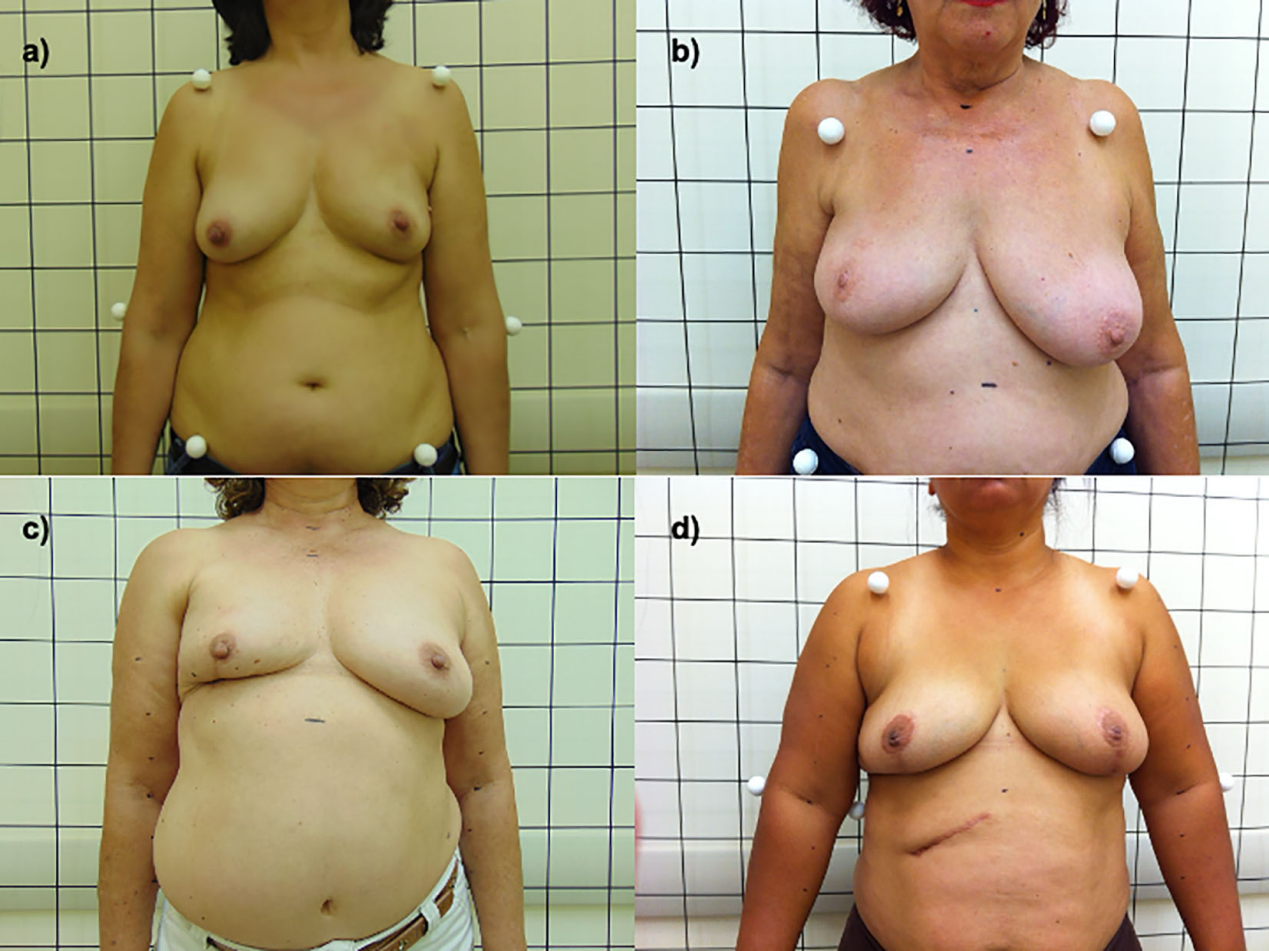
**(A)** concordant satisfaction; **(B)** discordant satisfaction; **(C)** concordant dissatisfaction; **(D)** discordant dissatisfaction.

Comparing the objective results of BCCT.core with the judgment of the patients, 77 (25.7%) were “concordant satisfaction”, 150 (50%) were “discordant satisfaction”, 56 (18.7%) were “concordant dissatisfaction”, 12 (4%) were “discordant dissatisfaction”, and 5 (1.7%) had no such evaluation (**Figure 1** exemplifies these four scenarios). Differences were observed in several general (EORTC-C30: physical, functional and emotional capacity, fatigue, pain, dyspnea, insomnia, and financial difficulties), specific breast (EORTC-BR23: body image, breast symptoms and side effects) and all BCTOS items. According to the same analysis (**Table 2**), the discordant dissatisfaction group had worse scores on physical, functional and emotional capacity and body image, with greater pain, insomnia, side effects, breast symptoms, functional symptoms and edema.

**Table 2:** Quality of life in relation to breast satisfaction status*

Questionnaire	Domain	Concordant satisfaction	Discordant satisfaction	Concordant dissatisfaction	Discordant dissatisfaction	p
EORTC	Global Health	80.95 (19.6)	79.33 (22.8)	72.92 (21.45)	70.83 (22.61)	0.096
QLQ C30	Physical capacity	85.97 (15.2)	79.29 (18.31)	73.42 (19.64)	72.22 (17.02)	<0.001
	Functional capacity	86.15 (23.48)	81.33 (26.12)	73.81 (29.1)	73.61 (24.06)	0.042
	Emotional Ability	66.99 (29.26)	65.59 (29.34)	45.83 (35.57)	43.75 (35.73)	<0.001
	Cognitive ability	71.43 (32.2)	70.67 (30.39)	61.9 (34.77)	58.33 (34.45)	0.178
	Social capacity	93.07 (15.85)	93.56 (17.88)	86.9 (22.41)	84.72 (30.53)	0.073
	Fatigue	18.47 (22.77)	18 (24.38)	32.34 (28.45)	31.48 (26.73)	0.001
	Nausea and vomiting	4.33 (10.95)	5.33 (14.1)	8.63 (19.07)	6.94 (16.6)	0.374
	Pain	24.03 (25.14)	30.78 (32.87)	36.61 (32.94)	50 (33.3)	0.018
	Dyspnoea	6.49 (17.13)	9.11 (22.81)	9.05 (33.55)	8.33 (15.08)	0.020
	Insomnia	22.51 (31.73)	30.67 (39.1)	34.52 (39.68)	55.56 (47.85)	0.027
	Appetite loss	6.49 (18.76)	10.22 (26.46)	15.48 (30.46)	18.18 (31.14)	0.177
	Constipation	19.48 (33.05)	24 (35.42)	21.43 (36.2)	30.56 (41.34)	0.679
	Diarrhoea	4.33 (18.22)	6.44 (21.4)	11.9 (27.29)	8.33 (28.87)	0.266
	Financial difficulties	15.15 (31.32)	13.78 (31.18)	28.57 (40.42)	13.89 (33.21)	0.038

EORTC	Body image	87.88 (21.61)	85.5 (20.96)	67.11 (34.1)	58.33 (40.2)	<0.001
QLQ BR23	Sexual functioning	75.54 (24.57)	77 (25.45)	70.24 (28.55)	72.2 (28.72)	0.403
	Sexual enjoyment	53.51 (28.52)	41.27 (30.36)	47.31 (28.25)	46.67 (38.01)	0.256
	Future perspective	59.31 (38.09)	56.89 (40.97)	46.43 (41.53)	47.22 (33.21)	0.240
	Side effects	20.04 (16.39)	23.33 (19.84)	33.67 (23.15)	34.13 (21.85)	<0.001
	Breast symptoms	16.77 (20.25)	17.78 (20.06)	28.57 (25.67)	34.03 (33.61)	0.001
	Arm symptoms	22.08 (23.95)	28.52 (28.83)	31.55 (28.41)	36.11 (35.81)	0.146
	Hair loss	28.43 (40.31)	22.02 (33.8)	36.78 (43.96)	38.1 (35.63)	0.339

BCTOS	Functional	1.64 (0.69)	1.76 (0.8)	2.05 (0.88)	2.38 (0.98)	0.002
	Cosmetic	1.82 (0.6)	2.21 (0.71)	2.78 (0.75)	2.68 (0.61)	<0.001
	Breast-specific pain	1.94 (0.87)	1.78 (0.8)	2.36 (0.99)	2.67 (0.89)	<0.001
	Oedema	1.41 (0.63)	1.59 (0.68)	1.83 (0.78)	1.88 (0.84)	0.003

*Mean and standard deviation.

Using the Bonferroni adjusted calculation (**Table 3**), comparing “concordant dissatisfaction” patients with “concordant satisfaction” patients, showed differences in the physical, functional and emotional capacity, fatigue, pain (QLQ-C30), body image, side effects, breast symptoms (QLQ-BR23) and in BCTOS functional capacity, cosmesis, pain and edema. Between the “concordant dissatisfaction” group and the “discordant satisfaction” group, there were differences in fatigue, pain and financial difficulties (QLQ-C30), breast symptoms (QLQ-BR23) and by BCTOS in the cosmetic and pain domains. In the “discordant dissatisfaction” patients compared to the “concordant satisfaction” patients, there were differences in the pain (QLQ-C30), body image (QLQ-BR23) and functional, cosmetic, and pain domains (BCTOS). In the comparison between “discordant satisfaction” and “discordant dissatisfaction” groups, differences in insomnia (QLQ-C30), body image (QLQ BR23) and pain (BCTOS). In the comparison between “concordant satisfaction” and “discordant satisfaction” patients, differences in physical capacity (QLQ C30) and cosmesis (BCTOS) were detected.

**Table 3:** Assessment of significant difference between groups using the Bonferoni corrected test.

Questionnaire	Domain	Different groups	p variables	p group
EORTC	Physical capacity	Concordant satisfaction x discordant satisfaction	0.046	<0.001
QLQ C30		Concordant satisfaction x concordant dissatisfaction	< 0.001	
	Functional capacity	Concordant satisfaction x concordant dissatisfaction	0.044	0.042
	Emotional Ability	Concordant satisfaction x concordant dissatisfaction	< 0.001	< 0.001
	Fatigue	Concordant satisfaction x concordant dissatisfaction	0.010	0.001
		Discordant satisfaction x concordant dissatisfaction	0.002	
	Pain	Concordant satisfaction x discordant dissatisfaction	0.045	0.018
		Concordant satisfaction x concordant dissatisfaction	0.017	0.018
		Discordant satisfaction x concordant dissatisfaction	0.048	
	Insomnia	Discordant satisfaction x discordant dissatisfaction	0.031	0.027
	Financial difficulties	Discordant satisfaction x concordant dissatisfaction	0.029	0.038
EORTC	Body image	Concordant satisfaction x discordant dissatisfaction	0.001	<0.001
QLQ BR23		Concordant satisfaction x concordant dissatisfaction	< 0.001	
		Discordant satisfaction x discordant dissatisfaction	0.002	
	Side effects	Concordant satisfaction x concordant dissatisfaction	0.001	<0.001
	Breast symptoms	Concordant satisfaction x concordant dissatisfaction	0.014	0.001
		Discordant satisfaction x concordant dissatisfaction	0.011	
BCTOS	Functional	Concordant satisfaction x discordant dissatisfaction	0.017	0.002
		Concordant satisfaction x concordant dissatisfaction	0.023	
	Cosmetic	Concordant satisfaction x discordant dissatisfaction	<0.001	<0.001
		Concordant satisfaction x discordant satisfaction	<0.001	
		Concordant satisfaction x concordant dissatisfaction	<0.001	
		Discordant satisfaction x concordant dissatisfaction	<0.001	
	Breast-specific pain	Concordant satisfaction x discordant dissatisfaction	0.040	<0.001
		Concordant satisfaction x concordant dissatisfaction	0.037	
		Discordant satisfaction x discordant dissatisfaction	0.004	
		Discordant satisfaction x concordant dissatisfaction	<0.001	
	Oedema	Concordant satisfaction x concordant dissatisfaction	0.003	0.003

## Discussion

Breast-conserving surgery involves tumor resection with free surgical margins and acceptable cosmetic results. Up to 30% of patients who undergo BCS require delayed repair due to unsatisfactory cosmetic results (8). This motivated the development of oncoplastic surgery, which also does not guarantee symmetry or satisfactory results (9). Nevertheless, patients who undergo breast conservation maintain higher QOL scores than patients who undergo mastectomy with and without reconstruction (6).

Our study included patients with a long follow-up period who were treated at a public tertiary cancer hospital that is part of the Brazilian Unified Health System, with a high rate of satisfaction with the cosmetic results (76.8%) after BCS in the self-assessment. Because we treat patients in the public system, we have a high number of patients with low education (63% with up to 8 years of schooling) (2), a fact that can impact values related to shame, concepts of body cosmesis, self-esteem, denial and non-questioning of the treatment offered (regardless of the outcome). Thus, there is a greater acceptance of cosmetic results, even if they are unsatisfactory, if there are favorable oncological results (10). In addition, medical teams are usually focused on cancer outcomes, which may undervalue cosmetic evaluation. Much of the information observed in this study derives from the results of the QOL questionnaire, when the patient was better heard and was able to express her opinion.

Patients with unsatisfactory cosmetic results had lower QOL scores compared to those with satisfactory results, a finding not observed for the correlation with the software evaluation. As shown in **Table 1**, patients with unsatisfactory results have worse conditions in 18/27 questions, which indicates that their overall QOL was poor. Unfortunately, we did not include anxiety/depression questionnaires or functional questionnaires (SPADI), which could broaden our assessment of dissatisfaction. When using the objective criteria of the software, only physical capacity, sexual pleasure, cosmesis and breast edema influenced the results, reinforcing the previously considered observations of anxiety and depression.

There is no one standard in the literature for the cosmetic evaluation of breasts after BCS. Historically, it is centered on the opinion of the patient or that of a panel of experts, which limits reproducibility, with uncertain validity because it is a subjective method. In this regard, BCCT.core, an objective and consistent tool, was used to evaluate these results, though showed low interobserver agreement (3, 4), which was also observed in our analysis. As demonstrated in a previous publication on this population, unsatisfactory results, from the patient’s point of view, were associated with younger age at diagnosis and the presence of larger tumors. According to the software, these results correlated with overweight at diagnosis, left-sided tumor, the presence of lymphedema, greater weight of the surgical specimen, surgical site infection, and a longer time interval between surgery and evaluation (2).

Several studies have found a linear relationship between cosmetic results and QOL. A study using the hospital anxiety and depression scale (HADS), body image questionnaire (BIQ), and Rosenberg self-esteem scale (RSE) revealed correlations between cosmesis, levels of anxiety/depression, body image, sexuality and self-care (11). Another study using the general health questionnaire (GHQ) found strong correlations between breast cosmesis and arm function and psychosocial function (12). In our study, we found that non-breast conditions can influence self-reported cosmesis, as the functional part and pain can negatively affect the evaluation of the patient. Therefore, it is necessary to understand that in the evaluation of the cosmetic outcome of the breasts, several factors can influence the negative results, which may be associated with patient, treatment or external factors (13–15).

Satisfaction with the cosmetic result and the assessment of QOL are individual and important components of a patient’s perception. The degree of satisfaction does not necessarily reflect the degree of symmetry because women with normal breasts may be dissatisfied with their breasts (16). Exner et al. attempted to correlate objective cosmetic outcomes using the breast analytical tool (BAT) with quality of life using the breast image scale and the EORTC QLQ-BR23 in 101 patients. They concluded that after BCS, the cosmetic outcome, through breast symmetry, is not an important factor for the QOL or self-esteem of patients (17). Similar findings were reported by Kim et al., who used BCCT.core for evaluation in 485 patients who underwent BCS (18).

Sneeuw et al. (12), in an evaluation of 76 patients, observed that the association between cosmetic and functional outcome and psychosocial health were stronger among younger patients and those treated longer. A recent study by Zwakman et al. (19) discussed the importance of evaluating cosmetic outcomes at long-term follow-up, as the current literature is limited in short-term outcomes. Using the QOL questionnaires of the EORTC, associated with objective (BCCT.core) and subjective (expert panel) cosmetic evaluations, 104 patients were evaluated with a mean follow-up time of 6.5 years, finding lower QOL in the presence of unsatisfactory cosmetic results. This corroborates our findings, as our series had a long follow-up (mean 7.4 years) and, as previously observed, a greater association of poor outcomes in young patients with longer follow-up times (2).

Unraveling the association between cosmetic results and QOL can be complex and difficult to interpret because the results can change throughout the patient’s lifespan, including values and not only by the surgical outcome. In addition, it is influenced by the type of questionnaire used, the socioeconomic and cultural profile of the patient, and the period of treatment in which it is being applied. It is necessary to consider the body changes over time, adverse effects of the treatment, initial expectations of the cosmetic result of the breasts, self-image body, sexuality and previous experiences, among other factors that are often underrepresented in these evaluations. Furthermore, QOL and cosmesis are subjective to be converted, objectively, into scales and compared. Either way, these are the current tools that we have at our disposal to continue patient-centered care and perform interventions when necessary (6).

Cosmesis and functional status were associated with better QOL. Similarly, breast tenderness, arm pain, limitation of movement were correlated with lower QOL because of the ability to maintain functionality (20). Our group of patients had a long follow-up period and a high rate of treatment-associated complications, which may have affected the results (7).

We sought to assess patient dissatisfaction, separating the type of dissatisfaction from the cosmetic results (**Tables 2**, **3**), because the major problem is when we have a discordant dissatisfaction. In this group, multiple conditions related to worse general quality of life and breast function were observed, which may correlate with worse functional aspects and depression. In addition, greater pain (QLQ-C30 and BCTOS), worse body image/cosmesis (QLQ-BR23 and BCTOS), and worse functionality (BCTOS). From the results observed, we can infer that low self-esteem and local symptoms, especially pain and functional limitations, negatively affect the cosmetic self-assessment of the patient. This is a novel finding that we should consider in the evaluation of our patients, especially when considering cosmetic surgery.

Limitations of this study include its retrospective, cross-sectional and uni-institutional nature, the nonuse of the more recent EORTC questionnaires, the Breast-Q and anxiety/depression questionnaires. In contrast, the use of widely used and reproducible questionnaires, associated with the considerable number of patients in long-term follow-up (longer than those previously reported), as well as objective and subjective cosmetic analysis, highlights the importance of this study.

## Conclusion

With the increase in breast cancer patient survival and patient-centered view, it is important to understand how the cosmetic results of the breasts influence QOL. We should listen to our patients, but it is essential to compare these results with objective, impartial and reproducible evaluations. An unsatisfactory cosmetic result after BCS is associated with a worse quality-of-life score, but other aspects, such as anxiety/depression, pain, and functional symptoms, may influence dissatisfaction and should not be overlooked.

## Data availability statement

The original contributions presented in the study are included in the article/supplementary material. Further inquiries can be directed to the corresponding author/s.

## Ethics statement

The studies involving humans were approved by Comitê de Ética em Pesquisa - Fundação Pio XII/ Hospital de Câncer de Barretos. The studies were conducted in accordance with the local legislation and institutional requirements. The participants provided their written informed consent to participate in this study. Written informed consent was obtained from the individual(s), and minor(s)' legal guardian/next of kin, for the publication of any potentially identifiable images or data included in this article.

## Author contributions

IO-J: Writing – original draft, Writing – review & editing. FB: Data curation, Writing – review & editing. AS: Supervision, Writing – review & editing. RV: Conceptualization, Funding acquisition, Writing – original draft, Writing – review & editing.
